# Assessing student engagement from facial behavior in on-line learning

**DOI:** 10.1007/s11042-022-14048-8

**Published:** 2022-10-24

**Authors:** Paolo Buono, Berardina De Carolis, Francesca D’Errico, Nicola Macchiarulo, Giuseppe Palestra

**Affiliations:** 1 Department of Computer Science, University of Bari ‘Aldo Moro’, Via Orabona 4, Bari, 70125 Italy; 2 Department Education, Psychology and Communication, University of Bari ‘Aldo Moro’, Via Crisanzio 42, Bari, 70122 Italy; 3 HERO srl, Rome, Italy

**Keywords:** Engagement measurement, Face behavior, Computer vision, LSTM

## Abstract

The automatic monitoring and assessment of the engagement level of learners in distance education may help in understanding problems and providing personalized support during the learning process. This article presents a research aiming to investigate how student engagement level can be assessed from facial behavior and proposes a model based on Long Short-Term Memory (LSTM) networks to predict the level of engagement from facial action units, gaze, and head poses. The dataset used to learn the model is the one of the EmotiW 2019 challenge datasets. In order to test its performance in learning contexts, an experiment, involving students attending an online lecture, was performed. The aim of the study was to compare the self-evaluation of the engagement perceived by the students with the one assessed by the model. During the experiment we collected videos of students behavior and, at the end of each session, we asked students to answer a questionnaire for assessing their perceived engagement. Then, the collected videos were analyzed automatically with a software that implements the model and provides an interface for the visual analysis of the model outcome. Results show that, globally, engagement prediction from students’ facial behavior was weakly correlated to their subjective answers. However, when considering only the emotional dimension of engagement, this correlation is stronger and the analysis of facial action units and head pose (facial movements) are positively correlated with it, while there is an inverse correlation with the gaze, meaning that the more the student’s feels engaged the less are the gaze movements.

## Introduction

Engagement plays a crucial role in the learning process [54], because it can influence the students’ retention, learning, test scores and graduation [1, 23]. Digital technologies offer the opportunity to attend online courses in order to gain access to education, despite spatial and temporal constraints. The COVID-19 pandemic changed dramatically education, with the distinctive rise of e-learning, whereby teaching is undertaken remotely, using digital platforms. Especially in online education student engagement is considered a necessary prerequisite for learning [27]. For this reason, the understanding of student engagement in this context is important to improve learning as well as teaching, since it might allow detecting problems with the course material, both in terms of content and presentation. Moreover, automatic engagement monitoring can be used in developing personalized learning environments able to tailor the experience on the student’s capabilities.

Student engagement has been defined in various ways, however scholars have largely identified it as a construct that contains three components: behavior, emotion and cognition [23]. Behavioral engagement regards the participation in an activity (i.e. the student completing an assignment), emotional engagement refers to students’ affective responses or feeling towards teachers, peers, the course and learning, whereas cognitive engagement refers to the task-specific thinking that a student employs while undertaking in an activity [32]. Note that these three components are not isolated processes but they are dynamically interrelated within the individual [23].

In online learning, the assessment of a student’s engagement can be done explicitly by asking the student to evaluate his/her perceived level of engagement in the lecture [24, 33], or implicitly, by capturing and analyzing data gathered from the student behavior [11, 13, 26].

The most common approaches to perform automatic engagement prediction are based on computer vision, which can be used to unobtrusively assess a student’s engagement by analyzing cues from facial expressions, head pose and gaze [47, 55]. These behavioral data can be detected through a camera, a low-cost device that is usually integrated into smartphones, computers, and other devices. Recently, the analysis of physiological signals captured from sensors (i.e. galvanic skin response, heart rate, EEG) is also being considered for estimating engagement [5, 34]. However, these sensors are more intrusive, since users must physically wear them, and also more expensive than a camera. For this reason in this research we focused on predicting people’s engagement from their facial expressions (facial action units), head pose, and eye gaze, using computer vision techniques and deep learning, and then we evaluated how the prediction was related to self-reported measures.

To develop a model to automatically predict a student’s engagement from facial behavior we look at the available datasets in this domain. The one used in the EmotiW 2019 challenge [19], whose goal was to predict the engagement intensity of a subject while he was watching an educational video (MOOC), was selected for two reasons: it is one of the few available that contains facial behavior recordings of people in the wild concerning distance education. Secondly, we had the possibility to compare the results of the approach proposed in this paper with those of the other research teams participating at the challenge.

The best results have been reached using Long Short-Term Memory (LSTM) networks because of their natural application to problems represented by temporal series [9, 44, 56]. Following this indications, we learned a model based on an ensemble of LSTM neural networks to better capture the impact of different groups of features denoting engagement from the face. In particular, three LSTMs were trained on different subsets of features: the gaze, the head location/rotation and the facial actions units. The result of each LSTM is combined using a fully connected layer to predict the engagement level in a scale from 0 to 1.

Then, in order to understand whether the learned model could be used in different e-learning environments, we collected new videos during an experimental study involving 30 students attending the first year of Computer Science degree. They attended to the learning session: “Introduction to IoT”. In order to elicit different engagement levels the lecture material was delivered according to two different formats: slides and video-lectures. During the learning session the students were video-recorded. At the end of each session each participant answered to a questionnaire for assessing his/her perceived engagement that, in the psychological literature, are strictly linked to the student’s level of skill and to the task’s difficulty (challenge) [3, 30]. Moreover, part of the questionnaire was devoted to investigate the “emotional engagement” that deals with learners’ emotional responses while learning.

In order to analyze collected videos, we developed a software aiming at analyzing video streams (recorded or real-time) with the focus on real-world conditions, such as various light conditions, spontaneous facial expressions, hands occluding the face. The software, using the learned LSTM model, provides a visual interface that visualizes the level of predicted engagement during the learning sessions.

Results show that behavioral measures took the same direction of the self-assessed measures since it emerged that they are generally higher in the video-lecture condition that, in our expectation, represented the most engaging condition. Indeed, the psychological subjective measures indicated that the students in our study felt themselves less engaged when interacting with the slides than with the video-lecture modality. Furthermore, the analysis of facial expressions (action units) and head movements revealed higher significant differences across conditions and they significantly contributed to the prediction of emotional engagement which is coherent with social signals research that stresses the importance of face in the emotional signal detection [48].

The rest of the paper is organized as follows: Section 2 presents the background and related work. In Sections 3 and 4 details of how to estimate student’s engagement with the proposed approach are presented. Section 5 illustrates the performed experiment with the discussion on the results. Conclusions and future work are drawn in the final section.

## Related work

Measuring and monitoring student engagement is relevant not only in traditional settings, such as physical classrooms, but also in other contexts such as e-learning, intelligent tutoring systems (ITS) [37, 49, 56], and MOOCs (Massive Open Online Courses) [18]. Engagement has been identified as one of the crucial variables fostering academic outcomes. It has been defined as the extent to which students feel actively engaged by their learning activities, e.g. thinking, talking and generally interacting with their peers and instructor [16]. Engagement can be considered more than just a behavioral aspect as “time and energy spent by the student” and, as in Handelsman’s definition [31], it is mainly composed by the affective investment of a person engaged in the on-line learning process [10]. The affective or emotional dimension of the engagement can be conceived as an experience of simultaneous occurrence of elevated concentration, interest, and enjoyment, closer to the so called ‘flow state’ that can be predicted by how much a task is challenging and also by the perceived skills [3]. According to the flow theory, the engagement is a balance of these two psychological processes, in the sense that the flow state is the combination of high perception of one’s own skills and feeling of challenge emerging from the task. Conversely, in case of low skills and challenge from an emotional point of view, students can feel apathy while, in case of high skills and low challenge, they can feel relaxed or bored and, in case of low skills and high challenge, anxious. The challenge-skill dynamics, and the ‘optimal’ flow state is also crucial for the learning process since it has been demonstrated that it has a positive impact on learning [54]. In this sense a finer-grained psychological understanding, which also includes the measurement of motivational and cognitive factors, can give a deeper understanding of the learning process and of its associated emotional engagement. Thus, it is of interest to endow a (human or virtual) tutor with the capability of both assessing and monitoring the student engagement, that is critical for successful teaching and learning, and relate these data with the quality of the materials and the student performance [31].

Traditional approaches that have been widely used for assessing a student’s engagement are based mainly on **self-reports**. They are usually collected using specific questionnaires in which students report their own perception of engagement, interest, boredom [30, 35, 46]. Self-reports are undoubtedly useful since and can also be used to relate quantitative data coming from sensors to the subjective perception of engagement. However, they have some limitations: they require time to be completed, they disturb the student during the learning process and they may have a bias depending on the interpretation that each student gives to the engagement state.

When learning through a digital environment, **automatic analysis** is necessary. New techniques enable the measurement of the engagement automatically. Video analysis [43] and physiological measures [3], such as Electroencephalogram (EEG), blood pressure, heart rate, or galvanic skin response, have been used to measure and monitor changes in learners’ mental state [22]. Especially in terms of engagement and alertness [10, 49], they are being used for detecting affect in this context. Even if the use of sensing technologies in learning environments provides valuable quantitative data about the mental state of the learner, which cannot be directly observable, they require specialized sensors and are difficult to be used in large-scale studies.

Many research works report how engagement can be estimated using **computer vision** techniques in which, unobtrusively, the student is monitored using a camera and cues from the face [43, 54], body posture and hand gestures are analyzed [4, 19, 38, 39].

Whitehill et al. [55] used **facial expressions** for automatic detection of students’ engagement in a classroom setting. Affective states pertaining to engagement such as Interest, Confidence, Excitement and Frustration were used to classify engagement in a four levels scale. At this aim, an SVM classifier has been used on facial features.

D’Mello, Chipman and Graesser [12] used student’s **posture** to discriminate between low engagement (boredom) and high engagement (flow). D’Mello and colleagues have investigated on the recognition of learning-centered affective states from facial expressions during interactions with tutoring systems [7, 16].

Bosch et al. [6] recognized various affective states of engagement such as boredom, engaged, concentration, confusion, frustration and delight in the real world settings. Features extracted from facial expressions and body movements were used to train a supervised classifier. Helme and Clarke investigated on typical behaviors in a classroom session of 40 minutes involving reading and problem-solving exercises of increasing difficulty levels [32]. They found that there was an increase in head and eye movements as time progressed, as well as with the increase of difficulty level. They also noticed a considerable occurrence of hand-over-face gestures during the session and for this reason developed a deep learning approach for automatic detection of hand-over-face gestures in images.

Engagement prediction from facial behavior has been considered also in [51] that compared human evaluation of the student engagement state with the one recognized by a model that considering facial expressions, head pose and eye gaze as features categorize the student state as engaged or distracted in a time slice of 10 seconds.

These studies not only indicate the importance of user engagement assessment in e-learning contexts, since this is useful in detecting mental states such as frustration, lack of interest and difficulty in understanding the content, but also the adequacy of approaches based on computer vision. The main advantage of using computer vision methods lies in the unobtrusiveness of the assessment process and ease of use, they simulate the classroom situation where a teacher observes whether a learner is motivated without interrupting his/her activities. However, several works analyze and recognize engagement from static images with individual subjects. In capturing behavioral cues or signals of engagement it is important to take into account the temporal dimension and the dynamic nature of this state. Therefore, it is necessary to study the pattern of user engagement across time, as in the case of on-line video lectures.

According to a recent work that focused on the automatic measurement and prediction of engagement from the analysis of facial expressions [39], head pose and gaze behavior, we decided to start from such features and to relate the predicted student engagement to the self reported one. This challenge is based on the EngageWild database [19]. The method proposed by [52] is similar to the one proposed in this paper and had the best performance among the team participating at the challenge (mean square error of 0.0597). After dividing each input video into overlapping segments, facial features were extracted with OpenFace [2] for each instance and, in addition, features were extracted also by using a pretrained convolutional neural network. Then a combinations of Long short-term memory (LSTM) and Fully connected layers were used to develop a model able to capture the temporal information and perform a regression on the engagement intensity. In the last step, fusions of models output was used to achieve better performance.

Another similar approach to the challenge was proposed in [53]. The videos were divided in several segments and, for each segment, a C3D pretrained model was used to extract features, eye gaze and head pose features were extracted with OpenFace and, with the help of OpenPose, body posture information was obtained. For each feature modality, a LSTM model followed by three fully connected layers was used and the results were ensembled by averaging the weights.

## Predicting students’ engagement from facial behavior

### The model

Measuring and predicting students’ engagement automatically from face videos is a challenging task because the engagement is not expressed in a unique way but through a set of different behaviors. As emphasized in 2, there are different facial expressions that characterize engagement. Moreover, it has to be recognized as a pattern evolving in time that characterize the student’s behavior during the learning process.

#### Dataset

Looking at available datasets of videos for learning the model from facial behaviors of students during e-learning activities, there are a few ones available such as DAiSEE [28] and EmotiW Challenge [19] datasets.

DAiSEE, is a multi-label video classification dataset composed of 9068 video snippets captured from 112 users in the wild. The dataset has four levels of labels indicating the intensity for each of the affective states of boredom, confusion, engagement, and frustration in the wild. Frame-by-frame annotation was accomplished by crowdsource labels for these four affective states. EmotiW 2019 Challenge is a dataset for common benchmarking and development of engagement assessment in different conditions “in the wild” (i.e. different background, illumination, poses, etc). It contains the subjects’ face videos when they are watching an educational video. The engagement levels of the face videos are divided into four states (from 0 to 3) where “0” indicates that the subject is completely disengaged and “3” indicates that the subject is highly engaged. All the engagement levels are labeled by five annotators and each video is labeled with an overall engagement intensity. The dataset contains 195 videos of variable length, recorded from 86 different subjects. It is divided in training set and validation set. The first one contains 147 videos of 59 different subjects and the second contains 48 videos recorded from 27 subjects.

In this paper, we focus on analyzing facial behavior features for engagement prediction [39] on the EmotiW 2019 Challenge dataset. This dataset was selected for two main reasons: first, this dataset is annotated in terms of engagement, not emotions; second, by participating to the challenge we had the possibility of comparing engagement prediction results with those of the other teams.

#### Learning the model

The main research methods used with this dataset includes frame-based, space-time-based features, and multi-modal features-based approaches.

The frame-based method uses a traditional manual method or a convolutional neural network (CNN) [42] to perform single feature extraction on 2D images and then use a feature fusion method to obtain video features [55].

The methods based on spatio-temporal features mainly use the Long Short-Term Memory (LSTM) to consider the variation of the pattern in time of the input and allow learning the temporal dynamics between the features as the mapping to engagement. LSTM are recurrent neural networks able to capture the different dynamics of time series and they have been shown to be efficient in sequence prediction problems. LSTM-based models have been successfully applied to engagement recognition using head movements [29, 41] and facial expression [17]. The winner of EmotiW 2018 challenge proposed a multi-instance learning framework that integrates traditional machine learning features from multiple perspectives with the LSTM network to determine the degree of engagement by improving the accuracy of the regression results [56]. In EmotiW 2019 challenge [19], the LSTM networks are suggested as effective in domains where temporal pattern have to be taken into account [9, 44, 56]. Temporal models such as LSTM show that temporal dynamics as well as the observation window and buffer delay are important factors to improve the performance of classifiers.

In order to extract features regarding facial behavior during e-learning, we used the Action Units (AUs) of the Facial Action Coding System (FACS) [20] together with the head pose and eye gaze features because, as it happens in classrooms, the movement and positions of the eyes and the head give clues about the interest of a student during the lecture [50].

There are very few freely available tools for extracting and tracking these features from videos. OpenFace 2.0 is an open source facial analysis toolkit capable of accurate facial landmark detection, recognition of a subset of Action Units, gaze tracking and head pose estimation [2].

The following 30 features were extracted from each frame in the video:

- EGD: x, y and z coordinates of the left and right eye gaze directions
- HLOC: the location of the head in terms of x, y and z coordinates
- HROT: the rotation of the head on x, y and z axis
- AUI: the intensity of 17 facial action units AU01_r, AU02_r, AU04_r, AU05_r, AU06_r, AU07_r, AU09_r, AU10_r, AU12_r, AU14_r, AU15_r, AU17_r, AU20_r, AU23_r, AU25_r, AU26_r, AU45_r
- AUP: presence of the facial action unit AU28_c

The selected AUs are all those that OpenFace can recognize. From each frame in a video, all such features are extracted and concatenated together to get individual temporal behavioral pattern. To this aim a deep learning approach based on Long Short-Term Memory (LSTM) network has been used.

**Fig. 1:**
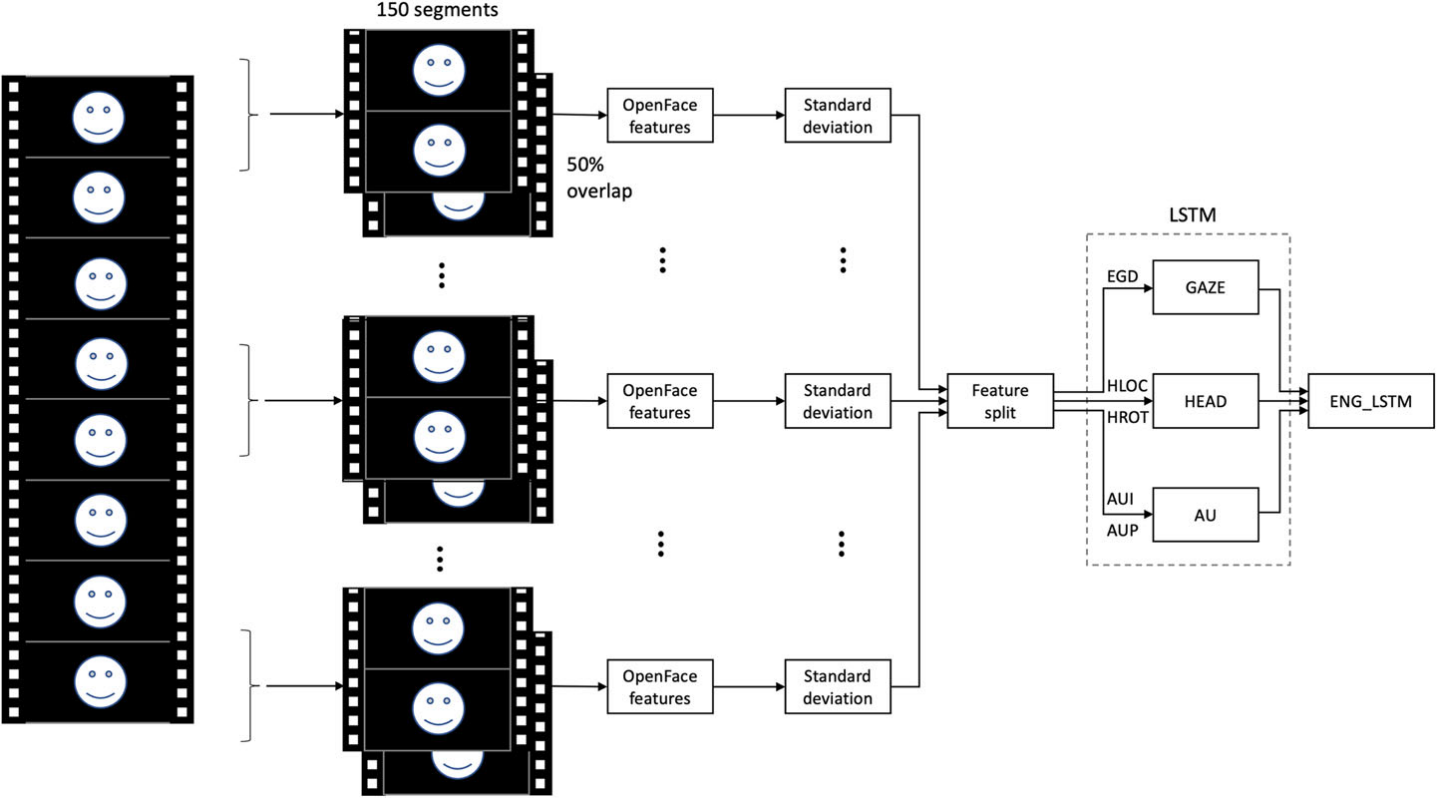
The architecture of the proposed model. Three LSTMs are trained on different subsets of data. The results of each network are combined with a fully connected neural network (ENG_LSTM)

In a first phase we trained a single LSTM network [14] then, according to the results of the first study, we revised the model and, in the new version presented in this paper, we used an ensemble of three LSTM networks, one for each group of features (Fig. 1).

Each LSTM network was trained on a different subset of features, in order to separately monitor the gaze (GAZE), the head pose/rotation (HEAD) and the facial actions units (AU). To combine the results of each LSTM network, we used three fully connected layers and the last one returns the intensity of the predicted engagement in the range [0,1]. Since in the videos of the dataset the final engagement level was expressed in a scale [0,3] we normalized it to [0,1] to be consistent with the output of our model.

In order to extract the same number of features from each video, having different lengths, we divided each video in 150 segments of variable length with an overlapping window of 50% and, for each of them, we calculated the standard deviation for each feature in each segment. The result of this pre-processing phase was then split into three subsets, each of them representing the input of the respective LSTM neural network (Fig. 1).

The first network (GAZE) was trained on the subset made by the features related to the gaze (denoted with EGD). This neural network is composed of 64 neurons in its LSTM layer. Then, two fully connected layers have been added and the last one returns the engagement intensity in the range [0,1]. The second network (HEAD) was trained on the subset of features related to the head location and rotation (denoted with HLOC and HROT). The architecture of this neural network consists of two stacked LSTM layers with 64 neurons each and three fully connected layers with 64, 32 and 1 neurons. Also in this network, the output of the last layer is the engagement intensity in the range [0,1]. The same structure was used for the third neural network (AU), which was trained on the subset composed by the features related to the 18 facial Action Units listed before (denoted with AUI and AUP). A further neural network (ENG_LSTM) is then used to combine the partial outputs of the three neural networks. This last network is composed by three fully connected layers with 16, 8 and 1 neurons. The final engagement intensity prediction is the output of the last layer of this fully connected neural network.

#### Model accuracy

The model validation and test have been done by participating in the EmotiW 2019 challenge, thus comparing our results with those of the other challenge participants. To be compatible with the accuracy measure of the challenge, the Mean Squared Error (MSE) has been selected as a loss function and Adam with the default parameters as optimizer [40].

The training was performed on 500 epochs. During the training, the best epoch reached a MSE of 0.04 on the training set and 0.079 on the validation set. On the provided test set, the result of our team (IntIntLab) is 0.077, far surpassing the baseline (0.15) [19]. The MSE value on the test set of the first three challenge participants was around 0.06, thus our approach is able to learn a model for analyzing the videos of the study participants with a good level of accuracy.

### The Engagement analysis tool

In order to provide to teachers, tutors and course designers with a tool for monitoring students engagement both in real-time or for analyzing recorded videos, we built EngANT (Engagement Analysis Tool), a software that, through a simple user interface shows the values of the extracted features, the engagement level, and the video frames that are being analyzed.

**Fig. 2:**
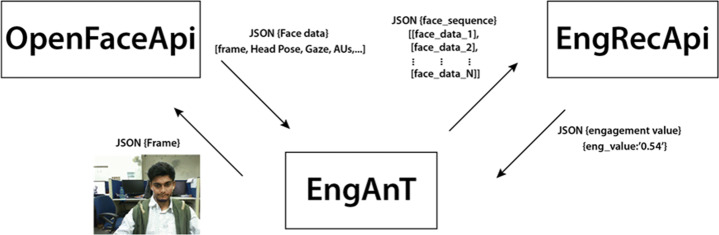
The EngANT Architecture Schema

**Fig. 3:**
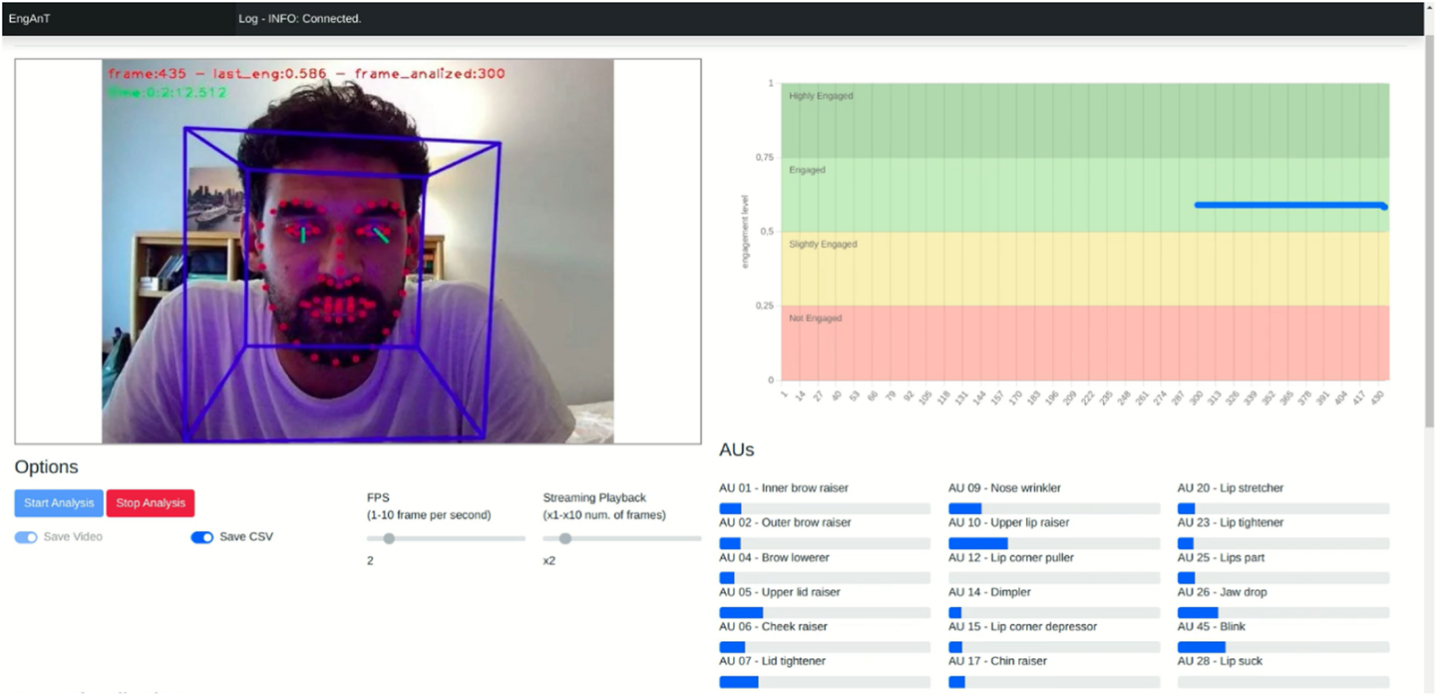
Interface of the software for analyzing engagement from video and in real time

As shown in Fig. 2, EngANT consists of three main components. *OpenFaceApi* is used to extract the features from the facial behavior; *EngRecApi* is a web service that provides an API that, given the features extracted during the video frame analysis, it returns the engagement prediction level; *EngANT* is a WSGI web server that runs a Flask application. It can be used through the user interface shown in Fig. 3 that handles the sessions of the engagement analysis. The components communicate through the correspondent APIs using JSON objects as input and output parameters.

As visible in Fig. 3 EngAnT User Interface is composed of four sections. The *Preview area* shows the live video with the indications of the current frame and time and a visual representation of the detected parameters is overlayed. The *Engagement level* (top right of Fig. 3), shows in four colored horizontal staked bars the levels of engagement. On the x-axis is represented the time, while on the y-axis is represented the level of engagement, frame by frame. The *Option* area (bottom left of Fig. 3) allows the user to change some options and to start/stop the analysis. The *Action Units* area (bottom right) shows the different detected AUs listed in Section 3.1. In Fig. 3 the highest detected AUs are AU 10 and AU 12, both related to lip position. At the top of the UI there is a dark horizontal status bar.

## The study

The study was performed at the Computer Science Department of the University of Bari, Italy, with the involvement of students attending the first year of the bachelor’s degree course of the same Department. The aim of the study was to test the quality of the model in predicting a student engagement level in relation to the subjective self-reported perception of engagement. Our on-line courses are supported by learning material in the form of slides and video presentations. Thus, we analyzed whether such formats elicited different levels of engagement.

### Data collection methodology

#### Participants

Data were collected from 31 healthy subjects. They were undergraduate Computer Science students (17 males, 14 females with a average age of 19 y.o.) that participated voluntarily in the study. Data about one male student that did not complete the entire session were discarded. Participants were randomly split into two groups, balanced according to gender, one for each condition. In total, data recorded from 30 participants were analyzed.

#### Materials

In order to select students interested in the lecture’s topic, we made a quick interview to 160 students of the first year in Computer Science, asking them to select a topic of their interest among a list of four: 1) Introduction to IoT, 2) Introduction to Computer Vision, 3) Introduction to Affective Computing, and 4) Introduction to Agents. The most preferred one was “Introduction to IoT” (85 preferences in total), then, according to this result, we designed and implemented the corresponding on-line lecture that was provided using two different presentation formats that are normally used in on-line education: slides and video-lecture (slides with the professor presenting in the video), with the aim to elicit different engagement conditions. We expected that, the video-lecture condition would be more engaging than the other experimental condition.

To measure the perceived engagement, we used a scale aimed at exploring game-based learning that has been created by translating and adapting the one proposed by [30] and the short-form of the UES (User Engagement Scale) questionnaire [45], were we skipped the questions about the evaluation of the application aesthetic and attractiveness. The adopted scale is then composed by the evaluation of four main dimensions of the flow theory [3]:

- engagement
- immersion
- motivational (challenge)
- cognitive (skills)

For each item, the assessment is done on a scale from 1 to 5, where 1 means “not at all” and 5 means “a lot”. The questions are listed in Fig. 1.

We performed a factorial analysis by extracting 3 factors which explained the 76% of variance (engagement factor: 57%, skill: 10%, challenge: 9%). In order to assess the reliability of data involved in the study we computed Cronbach’s alpha on sub-scales, which resulted all higher than 0.70 (values ranging from 0.7 to 0.9 are considered optimal [15]). From the answers to this questionnaire we calculated two indexes: the i) PEI - Perceived Engagement Index - calculated as the average of the questionnaire items and the ii) EEI - Emotional Engagement Index - concerning the answers to the part of the questionnaire for assessing the emotional dimension of engagement (Q2-Q4 in Table 1).

**Table 1:** Questionnaire Summary

Engagement	Q1. How interesting was the lesson?
	Q2. How much did you enjoy the lesson?
	Q3. How much following the lesson was engaging?
	Q4*. How much did you feel bored while following the lesson?
	Q5*. How much would you have preferred doing something else?
Challenge	Q6. How challenging was the experience?
	Q7. I felt challenged during the lesson.
	Q8*. How hard were you concentrating?
Skill	Q9. I felt up to the situation.
	Q10. I have exercised my skills to the fullest.
	Q11*. I didn’t feel very prepared during the lesson.

Questions with star (*) are reversed items in the statistical analysis

#### Procedure

The study procedure was structured as follows: upon the arrival at the laboratory, participants were informed about the purpose of the experiment and asked to read and sign a document explaining the experiment and a consent form to record their facial behavior. Participants, one at the time, seated on a comfortable chair in front of a computer with a Full HD 24 inch screen, a set of speakers, a mouse and a keyboard. Each participant watched at one lesson selected among the two different modalities (slides or video). The average time of each session was 9 minutes, which is considered the average attention time by students and the average duration time of a MOOC [36]. The participant’s behavior was captured by a webcam (Microsoft LifeCam Studio) that was placed on top of the monitor, to record the face and gaze behavior while interacting with the learning environment.

At the end of the session, participants were invited to complete the post-test questionnaire, described previously, in order to collect their perceived engagement. In total, 30 valid videos were collected (15 for each condition) which contained about 5 hours of recordings of facial behavior ready to be analyzed by our model.

#### Measuring engagement

To measure the engagement from the facial action units, head and gaze behavior, each collected video was analyzed using the model illustrated in Section 3 according to the pipeline in Fig. 1. In the same experiment also data from EEG signals using the Emotiv EPOC+ headset have been collected to be used for further analysis in the future. In order to be able to synchronize the video analysis to the resolution of the provided EEG data (0.1 Hz) we used a 10 seconds interval video analysis. The analysis started processing an initial segment of 10 seconds and then, the successive segments of 10 seconds each were added and processed until the end of the video. For each segment we obtained an engagement value in the range [0,1].

Table 2 reports an example of engagement measurement performed during the video analysis. The table shows key-points in a video analysis in which there is an estimation of change in the user engagement. In particular, the participant was following the lecture in the slide modality. Different facial behaviors during an experimental session are shown and the corresponding engagement value computed by our model. In the picture at the top, at the beginning of the interaction, the head and the gaze are straight in front of the screen and the model estimates a good level of engagement. Conversely, in the second picture, the participant is not looking at the screen and the estimated engagement is low (0.30). Afterwards, the participant is not looking at the screen and her eyes are closed, the face expresses mainly boredom and our model detects one of the lowest engagement values in the recorded session (0.28).

**Table 2:** The engagement intensity measured with the proposed LSTMs and the associated face in key points of the video

Time	Engagement	Frame
00:02:49	0.71	
00:05:22	0.30	
00:06:58	0.61	
00:08:00	0.28	
00:09:34	0.43	
00:12:27	0.44	

The model prediction, then, can be used to make the e-learning system aware of the student engagement and use this information to help instructors guide students in succeeding in a course or identify difficulties during the course [22], determine which activities and materials are more or less engaging and personalize content and feedback to the student, or help the instructor to identify low-engaged students during a course based on activities from that online course. Moreover, while the video stream is analyzed by the model, predictions about the level of engagement could help, for instance, the e-learning system to automatically provide motivation and advisory messages (i.e., asking about any difficulty) to motivate the student to increase their engagement. This would allow also to acquire feedback about the course and related materials.

## Results

In the analysis of the collected data we investigated the differences in the participants’ levels of engagement in the two proposed conditions.

Comparing the average of the self-reported engagement, the PEI, in the two conditions, on average the students felt more engaged with the video-lectures (*M* = 3.64) than in slides condition (*M* = 2.533). Due to the number of participants for each condition we performed the Wilcoxon test that indicated that the video-lectures condition was rated as more engaging than the slides condition (*p* = 0.00544). Such difference is significant at *p* < 0.01.

The same investigation was conducted on the two data sets representing the automatically estimated engagement in the two conditions. The mean values of the two sets of data reveals that the video condition (*M* = 0.69) have a higher value of estimated engagement than the slides condition (*M* = 0.61). Also in this case the difference is significant (*p* = 0.000279) at *p* < 0.01.

Since we analyze the entire video and the perceived engagement at the end of the lecture, the reported data and correlation is an ``1average” of the entire session. For this reason, we calculated the average of the minimum and maximum values estimated in the two conditions. Results are reported in Table 3

**Table 3:** Analysis of estimated engagement values in the two conditions

Condition	Average	Average min values	Average max values
Slides	0.61	0.42	0.728
Video-lecture	0.69	0.61	0.737
Wilcoxon *p* < 0.01	*p* = 0.000279	*p* = 0.00064	*p* = 0.215

These data indicate a clear difference in the minimum values of engagement between the two conditions, which means that while on average the difference is not so high, in the slide condition the engagement reaches statistically significant lower values than in the video-lecture condition.

The Pearson correlation between the engagement detected automatically from the face and the one coming out from self-reports reveals that the facial behavior is weakly positively correlated to the PEI (*ρ*= 0.39), that is, the more engagement is expressed, the more engagement is perceived.

Since facial expressions are always related to emotions and affective states, we also calculated the correlation between the answers to the section of the questionnaire concerning the emotional dimension of engagement (the EEI) and the data automatically detected by the facial behavior analysis module. The Pearson correlation was used again to this purpose and its results show that there is a weak correlation between the EEI and global facial behavior (ENG_LSTM, *ρ*= 0.38). The results of the different analyses of the facial behavior components reveal a difference in the correlation of positive and negative component of EEI. In particular, in case of positive emotional experience, the highest correlation is with the facial Action Units behavior (ENG_LSTM_AU, *ρ*= 0.37) and a weak inverse correlation with the gaze behavior (ENG_LSTM_GAZE, *ρ*=-0.36). While, in case of self-reported negative emotional experience, a weak inverse correlation emerges, in case of self-reported negative emotional experience with the detection from the ENG_LSTM_AU (*ρ*=-0.39) and the head behavior (ENG_LSTM_HEAD, *ρ*=-0.35), as shown in Table 4.

**Table 4:** Correlation between EEI and Facial Behavior (Global, AU, Gaze and HeadPose)

	Global	AU	Gaze	Head
EEI	0.38	0.37	− 0.32	0.24
PosEEI	0.36	0.37	− 0.36	0.15
NegEEI	− 0.40	− 0.39	0.30	− 0.35

According to such findings, the facial behavior contributes mainly in the higher engaging condition starting from the hypotheses (video-lecture condition), showing that the learning format can significantly contribute to students’ engagement [11].

In order to make such results stronger, the sample size should be enlarged. Moreover, according to the results of the questionnaire, participants reported high levels of skills and low levels of challenge. According to the Flow theory, they were less likely to be engaged [3]. Thus, the learning tasks should be more challenging, in order to better capture both physiological and psychological engagement.

## Conclusions and future work

In this work we proposed a study aiming at predicting student’s engagement automatically in the context of on-line learning from continuous video streaming. The study goal was two-fold: on one side we wanted to understand the efficacy of the proposed approach for automatic prediction of engagement from facial expressions, head pose and gaze movements and compare it with the perceived level of engagement; in addition, we wanted to analyze the engagement tendency in the learning activity performed using two different modalities: slides and video-lecture (lecture with a teacher).

To this aim we performed an experiment aiming at collecting videos of students while attending an on-line course in these two modalities and their self-reports about perceived engagement, that in the psychological literature are strictly linked to the students’ level of skill and to the task’s difficulty (challenge) [3, 30]. In order to analyze the collected videos we developed a tool that, using a model based on an ensemble of LSTM neural networks, predicts the level of engagement from facial expressions, gaze behavior and head pose. Then, we looked at possible correlations with self-reported data.

The psychological measures indicated how students in our study felt high levels of skill and less level of tasks’ challenge mainly in the slide condition. This evaluative pattern influenced their levels of perceived engagement, by inducing them to feel engaged mainly on video-lectures. Furthermore, results showed that each learning format can elicit different levels of engagement by contributing to give, together with subjective measures, a more structured framework.

An interesting finding concerns the emotional dimension of engagement, since this dimension is more related to facial behavior. In the future, we plan to better investigate on such aspects in our experimental design and analyze how specific emotions (such as the cognitive ones), can be recognized from facial behavior during on-line lectures and how they contribute to improve the engagement estimation [11].

Despite the novelty of the present work and our findings, there are several limitations that should be overcome. In order to reinforce our conclusions, the sample size should be enlarged and differentiated respectively by the level of perceived skill or challenge, because the considered sample was unbalanced in favor of the first dimension.

As future work, to overcome the problem that the questionnaire is filled in by the participants a the end of the entire lecture, while prediction is made every 10 seconds, we plan to asking expert human raters to manually annotate the videos with engagement levels. Moreover, we aim at comparing the measurement of the engagement from the face with the one from physiological data taken from various sensors (EEG, Galvanic Skin Response and Heartbeat). In this direction, during the study presented in this paper, we already collected data through a BCI using the EMOTIV EPOC+ headset and we will perform the analysis of these new data to understand whether this modality provides different insights on the student mental state [8, 25]. Other less intrusive sensors can be considered such as those detecting ECG, that can be worn on the chest or arm, such as in [21], where a chest sensor (Polar OH 10) has been used to monitor the user workload.

Another interesting investigation to perform would be the assessment of contents provided during the lecture in order to understand the relation between the measured engagement and the efficacy of the presentation material.

In summary, the automatic real-time measurement of engagement can help to monitor and understand, in a more refined way, in which part of the learning session the learners are more or less engaged, considering also their psychological processes related to a particular task and, therefore providing a support to course re-design, human and virtual tutors.

## Data Availability

The dataset generated and analysed during the current study are not publicly available because participants gave the permission for recording and data computation only, not sharing raw data but are available from the corresponding authors on reasonable request.
